# Machine Learning-Driven Muscle Fatigue Estimation in Resistance Training with Assistive Robotics

**DOI:** 10.3390/s25216588

**Published:** 2025-10-26

**Authors:** Jun-Young Baek, Jun-Hyeong Kwon, Hamza Khan, Min-Cheol Lee

**Affiliations:** 1 Department of Mechanical Engineering, Pusan National University, 2, Busandaehak-ro 63beon-gil, Geumjeong-gu, Busan 46241, Republic of Korea; 2 Ronfic. Co. Ltd., Busan 48058, Republic of Korea

**Keywords:** muscle fatigue estimation, resistance training, assistive robotics, machine learning, isokinetic exercise

## Abstract

Monitoring muscle fatigue is essential for ensuring safety and maximizing the effectiveness of resistance training. Conventional methods such as electromyography (EMG), inertial measurement units (IMU), and ratings of perceived exertion (RPE) involve complex procedures and have limited applicability, particularly in unsupervised or robotic exercise environments. This study proposes a machine learning-based approach to directly predict RPE from force–time data collected during repeated isokinetic bench press sets. Thirty-two male participants (64 limb datasets) performed seven sets at a standardized 7RM load, with load cell data and RPE scores recorded. Biomechanical features representing magnitude, variability, energy, and temporal dynamics were extracted, along with engineered features reflecting relative changes and inter-set variations. The findings indicate that RPE is more closely related to relative fatigue progression than to absolute biomechanical output. Incorporating engineered features substantially improved predictive performance, with the Random Forest model achieving the highest accuracy and more than 93% of predictions falling within ±1 RPE unit of the reported values. The proposed approach can be seamlessly integrated into intelligent resistance machines, enabling automated load adjustment and providing substantial potential for applications in both athletic training and rehabilitation contexts.

## 1. Introduction

Regular physical activity is widely recognized for its numerous benefits, including enhanced cardiovascular health, prevention of chronic diseases, improved physical performance, and overall well-being [1,2,3,4]. However, improper exercise techniques or excessive training loads can significantly increase the risk of injury [5]. Among various risk factors, muscle fatigue plays a particularly important role, as it accumulates progressively during repeated activity, potentially exceeding the tolerance of muscle tissues and leading to musculoskeletal disorders (MSDs) and performance decline [6,7]. Therefore, early detection and accurate prediction of muscle fatigue are essential to ensure safety and optimize training outcomes in both sports and rehabilitation contexts [8,9,10].

Timely fatigue assessment in sports training enables dynamic adjustments of exercise intensity, reducing the risk of overtraining and injury [11]. In rehabilitation, patients often perform prescribed exercises without direct supervision, increasing the likelihood of incorrect execution or excessive loading, which may result in secondary injuries [12]. Thus, quantitative monitoring of fatigue progression provides valuable feedback to support safe and effective exercise in both professional and clinical environments [13,14].

Recent advances in wearable robotics have expanded their applications from industrial and mobility support to strength training and rehabilitation [15,16,17]. In such robotic exercise systems, fatigue detection is crucial for adaptive control, allowing automatic adjustment of resistance according to the user’s physical state [18,19]. However, methods for accurately predicting exercise-induced fatigue and integrating them into robotic control systems remain underdeveloped [20,21].

Most existing approaches treat fatigue estimation as a binary classification problem, distinguishing only between “fatigued” and “non-fatigued” states [22,23]. This oversimplifies the inherently continuous nature of fatigue and limits the ability to detect subtle transitions for early intervention. Furthermore, many models are task-specific, limiting their generalizability and scalability across various exercise modalities [24,25].

**Figure 1:**
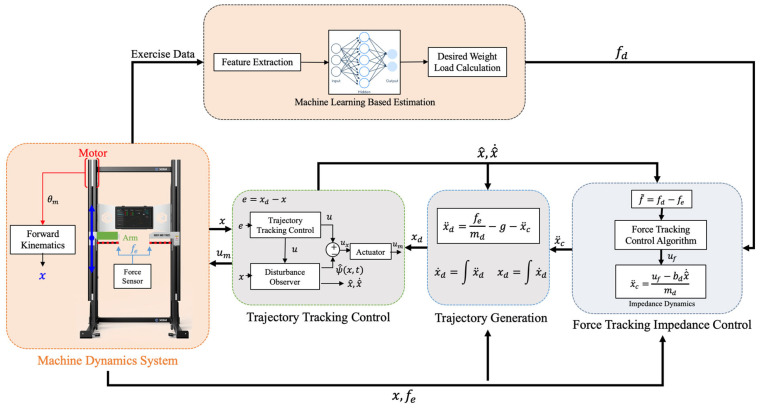
Configuration of XIM control system. Orange areas indicate the modules where data is received and machine learning is applied for decision-making, while gray areas represent the logic responsible for force control.

To address these limitations, this study proposes a multi-level fatigue estimation approach that uses the Rating of Perceived Exertion (RPE) as a validated and practical reference for labeling fatigue states. RPE is strongly correlated with objective physiological indicators such as sEMG, heart rate, and velocity loss, making it particularly suitable when specialized equipment is unavailable or impractical. In this work, force–time data collected from a load cell during bench press exercises using the XIM isokinetic robotic system (Ronfic Co., Ltd. Busan, Republic of Korea ) were processed to extract biomechanical features. These features were then paired with RPE scores to train machine learning models capable of predicting fatigue on a continuous scale, rather than binary classification. The proposed method is designed for integration into the XIM control system (Figure 1), enabling adaptive load modulation to improve both safety and training effectiveness in robotic resistance exercise.

While integrating multiple biosignals such as electromyography (EMG), heart rate (HR), and motion capture data can offer a more comprehensive understanding of neuromuscular fatigue, the present study prioritized real-world applicability. Unlike laboratory settings that rely on complex instrumentation, practical resistance training environments require lightweight, non-invasive, and easily deployable sensing solutions. Therefore, force–time data from the load cell sensor embedded in the resistance training device were utilized, allowing fatigue estimation without additional equipment or intrusive procedures. Moreover, EMG signals require precise electrode placement and are prone to interference from sweat and motion artifacts, which limits their reliability during dynamic exercise. Accordingly, force–time-based estimation provides a more feasible and robust approach for continuous fatigue monitoring in applied training contexts.

## 2. Related Works

A wide range of studies have been conducted to accurately assess and predict muscle fatigue, most of which combine sensor-based data acquisition with data-driven modeling techniques.

One of the most widely used approaches involves surface electromyography (sEMG), which measures the electrical activity of muscles and provides direct insight into fatigue progression [12,22,23,24]. For instance, Chattopadhyay et al. [23] extracted a comprehensive set of time- and frequency-domain features from sEMG signals and analyzed inter-subject and inter-trial variability in fatigue patterns. Dong et al. [24] combined sEMG data with accelerometer measurements to evaluate whole-body fatigue and proposed a “forgetting factor” and a “fatigue fusion coefficient” to integrate localized muscle fatigue estimates into a global fatigue index. Despite their effectiveness, sEMG-based approaches face several practical limitations, including sensor detachment during dynamic exercise, susceptibility to motion artifacts, and restricted spatial coverage that limits measurements to the specific muscle where the sensor is placed.

To address these limitations, recent studies have increasingly adopted wearable sensors such as inertial measurement units (IMUs) and load cells, which are lightweight, non-invasive, and capable of capturing continuous biomechanical data in real-world environments [25,26,27,28]. Jiang et al. [29] introduced a data-driven fatigue prediction approach by combining wearable IMUs and a force plate to estimate self-reported fatigue levels during three types of exercises: squat, high knee jack, and corkscrew toe-touch. They employed both Random Forest (RF) and Convolutional Neural Network (CNN) regression models, and CNN consistently achieved higher correlations with fatigue ratings, with center of pressure (COP) displacement identified as a key predictive feature. Marotta et al. [30] employed multiple IMUs to detect running-induced fatigue and demonstrated that combining kinematic features from lower-limb segments improved model accuracy compared with single-sensor configurations, particularly under subject-independent validation conditions. Stohrmann et al. [31] further analyzed kinematic changes induced by fatigue using wearable sensor data, demonstrating that these methods can reliably capture temporal characteristics of movement during walking, running, and sprinting.

Although fatigue is fundamentally a physiological process involving metabolic and neuromuscular mechanisms, numerous studies [26,27,29] have shown that these internal changes are reflected in alterations of movement kinematics. Fatigue-related reductions in neuromuscular control can lead to increased movement variability, decreased smoothness, and altered coordination patterns—all of which can be effectively captured by IMU-derived motion features. Accordingly, IMU-based measurements serve as indirect but practical indicators of fatigue-induced motor behavior changes, providing a non-invasive means to infer physiological fatigue when continuous biosignal monitoring is impractical or intrusive.

Beyond these kinematic approaches, biomechanical and neuromuscular features extracted from force–time data have also been extensively investigated to characterize muscle fatigue, particularly during isometric contractions. Dalton et al. [32] demonstrated that ramp maximal voluntary isometric contractions (MVICs) impair early rapid torque generation and muscle activation to a greater extent than rapid MVICs, despite comparable levels of peripheral fatigue. Similarly, Lin et al. [33] reported that fatigue suppresses both the amplitude of ideal force trajectories and gamma-band oscillations during rhythmic gripping tasks, suggesting a disruption in neuromuscular recalibration.

These studies collectively highlight that fatigue induces measurable alterations in force output characteristics and motor control dynamics, which are crucial for understanding perceived exertion during resistance exercise.

Furthermore, these biomechanical and neuromuscular descriptors provide valuable insight for feature selection, as they represent physiologically meaningful variables that can enhance the robustness and interpretability of machine learning models estimating fatigue or RPE.

In addition to direct physiological measurements, the Rating of Perceived Exertion (RPE) has been widely validated as a practical and scientifically robust indicator for fatigue estimation. Lea et al. [10] investigated the relationship between the Borg CR-10 RPE scale and physiological indicators such as sEMG, heart rate (HR), and blood lactate concentration (BLa) during resistance training. Their findings showed strong positive correlations (r ≥ 0.80) between RPE and all three physiological measures, with RPE responding sensitively to changes in exercise load. Zhao et al. [34] examined the relationships between RPE, velocity loss, and the sEMG-based Spectral Fatigue Index (SFI) during back squat exercises. Their findings revealed a significant positive correlation between RPE and SFI, while no significant association was observed between velocity loss and SFI. These results suggest that RPE may serve as a reliable indicator of muscle fatigue during non-explosive lower-body resistance exercises, whereas velocity loss may not be suitable in such contexts.

Despite these advancements, existing approaches still face several limitations. Many studies have focused on specific exercise modalities or sensor configurations, which limits their generalizability across diverse training contexts. Moreover, most predictive models have been developed as offline analytical tools without direct integration into adaptive control systems for robotic resistance training. This lack of integration reduces their applicability in real-world scenarios, where continuous fatigue estimation is essential for dynamic load adjustment and safety.

Building on these previous findings, the present study adopts RPE as the reference label for fatigue state estimation and utilizes force–time data collected from a load cell during isokinetic bench press exercises. Biomechanical features extracted from these data are paired with RPE values to train a machine learning model capable of multi-level fatigue prediction. Unlike existing studies, this work is designed for seamless integration into a robotic resistance training system (XIM, Ronfic Co., Ltd. Busan, Republic of Korea), providing a foundation for adaptive load modulation and enabling the development of more effective and personalized training strategies.

## 3. Materials and Methods

### 3.1. Experimental Approach to Problem

This study employed a data-driven methodology to develop a resistance adjustment system capable of automatically evaluating force profiles during bench press exercises using a single regression model. The bench press protocol was selected as a standardized exercise to induce muscle fatigue under controlled laboratory conditions, ensuring consistent and repeatable measurements. Force–time data were collected from participants performing consecutive sets, allowing for the analysis of fatigue progression and the identification of key biomechanical indicators associated with exertion levels.

The extracted features from these force profiles—including magnitude, variability, and temporal dynamics—were then used to train and validate a single regression-based predictive model designed to estimate continuous fatigue levels rather than simply classifying binary states. This approach enables more precise modeling of fatigue as a progressive phenomenon and supports the development of adaptive resistance strategies based on quantitative biomechanical feedback.

### 3.2. Participants

The experimental protocol was reviewed and approved by the Public Institutional Review Board of the Ministry of Health and Welfare (Approval No. P01-202410-01-054). Written informed consent was obtained from all participants before the commencement of testing. Thirty-two healthy male participants (mean ± SD: age = 33.2 ± 5.87 years; height = 172.84 ± 4.62 cm; body mass = 74.4 ± 11.58 kg) were recruited. Inclusion criteria were as follows: (1) at least two years of recreational resistance training experience, (2) regular engagement in bench press exercises two to three times per week, and (3) no history of upper-body injuries that could impair performance. Each limb was analyzed independently, resulting in a total of 64 limb datasets from the 32 participants.

### 3.3. Procedures

**Figure 2:**
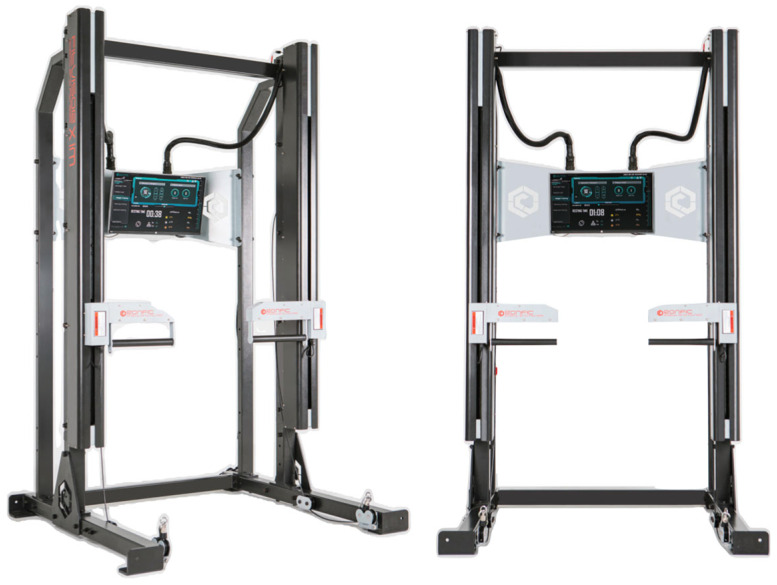
The system includes an integrated load cell for force measurement and an electronic resistance adjustment unit.

**Figure 3:**
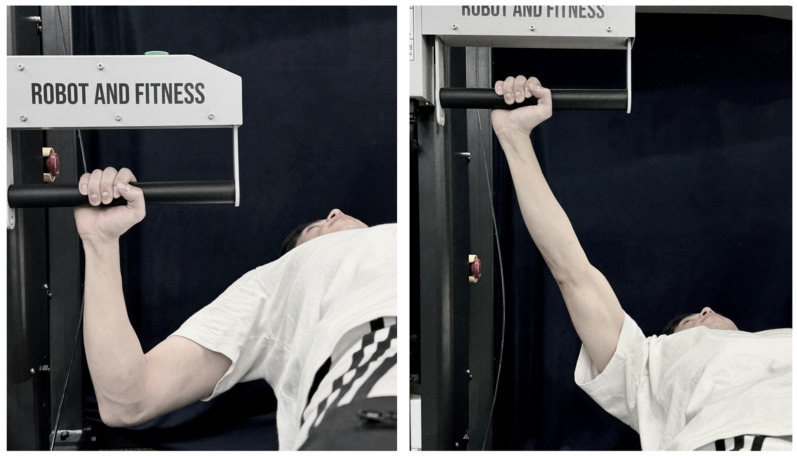
Bench press movement phases used in the study: starting position with barbell aligned over the mid-chest (**left**); concentric contraction from the starting position to full elbow extension (**right**).

All testing was performed on a modified, isokinetic-based Smith machine as shown in Figure 2 (XIM Machine, Ronfic Inc., Busan, Republic of Korea). The machine was calibrated according to the manufacturer’s specifications prior to data collection. The bench press range of motion (RoM) was standardized for each participant as shown in Figure 3:

1. Participants adopted a supine position with the grip bar aligned over the mid-chest.
2. The bar was lowered to the participant’s preferred bottom position, ensuring a minimum elbow flexion angle of 90°, and held for three seconds.
3. This position was registered as the starting point for all trials.

Following a warm-up session at a velocity of 0.7 m/s, the main protocol consisted of 7 sets of 8 repetitions performed at a velocity of 0.3 m/s to induce fatigue. Each repetition involved a concentric phase from the starting position to full elbow extension, followed by an eccentric phase returning to the start position in Figure 3. Eccentric loads were adjusted to each participant’s self-reported 7-repetition maximum (7RM) using the machine’s electronic resistance adjustment system. Rest intervals of two minutes were provided between sets. Testing concluded after the completion of the seventh set.

### 3.4. Data Collection and Preprocessing

Participants were instructed to perform maximal-effort bench press repetitions under a structured protocol. Each set consisted of eight repetitions executed at the participant’s maximum effort. A total of seven sets were performed to capture meaningful changes in both force output and perceived exertion as fatigue progressed.

The experimental sequence was as follows:

1. Warm-up—Participants performed bench press at a velocity of 0.7 m/s to ensure proper form and adaptation to the equipment.
2. Main exercise—Bench press at a controlled velocity of 0.3 m/s to induce fatigue, executed with the participant’s self-reported 7-repetition maximum (7RM) load.
3. RPE assessment—Immediately after each set, participants reported their exertion level using the OMNI Resistance Exercise Scale (OMNI-RES, 0 = “extremely easy” to 10 = “extremely hard”).
4. Rest—Participants rested in a seated position for two minutes before starting the next set.

**Figure 4:**
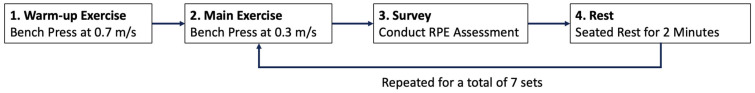
Experimental protocol for repeated bench press performed for data collection.

This cycle was repeated for all seven sets, ensuring synchronized collection of load cell-based force data and RPE scores for subsequent feature extraction and model training as shown in Figure 4.

The experimental dataset consisted of raw force signals sampled at 20 ms intervals (50 Hz). Only the push phase of the exercise was extracted, and separate files were generated for the left and right limbs of each participant. Both raw and processed data subsequently underwent a series of preprocessing steps to ensure suitability for machine learning analysis.

Continuous force sequences were divided into individual repetitions by identifying the onset and offset of each push motion, allowing each repetition to be independently analyzed and labeled with the corresponding fatigue level. To reduce high-frequency noise, a fourth-order Butterworth low-pass filter with a cutoff frequency of 20 Hz was applied to the raw signals.

To account for inter-individual variability in absolute force magnitudes, signals were normalized by dividing the measured force (*F*) by body mass (*m*). In addition, an allometric scaling approach was applied to better capture the nonlinear relationship between muscle strength and body size, where normalized force (*F_N_*) was calculated as

(1) $$F_{N}=\frac{F}{m^{0.67}}$$

### 3.5. Feature Extraction

To quantitatively represent the biomechanical and neuromuscular characteristics of each repetition, a comprehensive set of descriptive features was extracted from the normalized force–time signals. The primary objective of feature extraction was to transform raw sensor measurements into meaningful indicators that reflect various aspects of muscle function, including maximal strength, force stability, contraction dynamics, and energy expenditure, thereby enabling the continuous estimation of fatigue levels with a single regression model. Magnitude-related features such as peak force, mean force, and root mean square (RMS) capture the overall intensity and sustained output of muscle contractions, while variability and distribution metrics including variance, standard deviation, skewness, and kurtosis quantify the stability and shape characteristics of the force profile. Temporal descriptors such as time to peak force and rate of force development (RFD) provide insight into the dynamics of neuromuscular activation and explosive capability. Energy-based features including work, the work-to-standard deviation ratio, and the composite measure of peak × RMS represent the cumulative mechanical load and efficiency of muscle contractions, whereas composite indicators such as the Δ × ratio integrate multiple statistical dimensions to enhance the discriminative power across different fatigue states as shown in Table 1. By combining these heterogeneous features into a unified representation, the resulting dataset captures a comprehensive and multidimensional profile of muscle performance and fatigue progression, and these features serve as critical inputs for training the regression model to predict fatigue as a continuous variable with improved stability and generalizability.

Feature Extraction. (i) Primary Biomechanical Features (ii) Primary descriptors were derived from each set-level force profile, including.

Peak Force:

(2) $$F_{max}(s)=\underset{t\in s}{\max}\;f(t)$$

Mean Force:

(3) $$\bar{F}\left(s\right)=\frac{1}{N}\sum_{i=1}^{N}f_{i}(s)$$

Root Mean Square:

(4) $$RMS\left(s\right)=\sqrt{\frac{1}{N}\sum_{i=0}^{n}{f_{i}(s)}^{2}}$$

Variance and Standard Deviation:

(5) $$\sigma_{f}^{2}=\frac{1}{N-1}{\sum_{i=0}^{n}\left(f_{i}\left(s\right)-\bar{F}(s\right))}^{2}$$

Skewness and Kurtosis:

(6) $$Skew\left(s\right)=\frac{\frac{1}{N}{\sum_{i=0}^{n}\left(f_{i}\left(s\right)-\bar{F}(s\right))}^{3}}{\sigma^{3}},\;Kurt\left(s\right)=\frac{\frac{1}{N}{\sum_{i=0}^{n}\left(f_{i}\left(s\right)-\bar{F}(s\right))}^{4}}{\sigma^{4}}$$

Work:

(7) $$W\left(s\right)=\sum_{i=1}^{n}f_{i}\left(s\right)∆t$$

Time to Peak Force (TTP):

(8) $$TTP\left(s\right)=arg\underset{t}{\max}f(t)-t_{start}$$

Rate of Force Development (RFD).

(9) $$RFD\left(s\right)=\underset{t}{\max}\frac{∆f(t)}{∆t}$$

These features are well-established indicators of neuromuscular output and variability under fatigue conditions.

To explicitly encode fatigue progression, secondary features were computed as relative changes across sets, incorporating baseline-referenced and previous-set-referenced differences and ratios. Additional logarithmic, exponential, and interaction terms (e.g., Work/SD, Peak × RMS) were engineered to amplify discriminative signals observed during exploratory analyses. Set index was included as a numeric feature to model longitudinal trends in exertion.

Baseline-relative descriptors (first set as individual reference):

(10) $$∆f_{base}\left(s\right)=f\left(s\right)-f\left(1\right),\rho_{f,\;base}\left(s\right)=\frac{f(s)}{f(1)}$$

These normalize absolute inter-individual strength differences, focusing instead on relative decline from initial capacity.

Previous-set-relative descriptors:

(11) $$∆f_{prev}\left(s\right)=f\left(s\right)-f\left(s-1\right),\;\;\;\;\rho_{f,\;prev}\left(s\right)=\frac{f(s)}{f(s-1)}\;\;\;\;\;\;\;$$

These features capture acute fatigue accumulation and partial recovery dynamics between successive sets, providing essential information for modeling continuous fatigue progression. Prior to model training, feature preprocessing was performed to ensure data quality and maximize predictive performance. Non-numeric variables, constant features, and those with excessive missing values were removed from the dataset, and remaining missing entries were imputed using median values where necessary. To identify the most informative predictors, Pearson correlation analysis was conducted against the RPE labels, and features with an absolute correlation coefficient (|r|) of 0.5 or higher were retained as high-signal candidates. The selected features were then standardized to a common scale to ensure stable optimization and consistent learning behavior in the single regression model, enabling it to effectively capture the relationship between biomechanical indicators and perceived exertion levels.

### 3.6. Training and Analysis

Given the nonlinear, noisy, and subject-dependent nature of fatigue-related force–time signals, three representative regression models—Random Forest (RF), HistGradientBoosting (HistGBR), and Ridge Regression (RR) [35,36,37,38]—were systematically evaluated to determine the most effective predictive approach for estimating ratings of perceived exertion (RPE). These models were selected to represent distinct methodological paradigms: ensemble bagging (RF), boosting (HistGBR), and regularized linear regression (RR). This comparative analysis aimed to capture a broad spectrum of feature–target relationships while assessing the trade-offs between model complexity, interpretability, and generalization performance.

The selection of regression models was guided by evidence from previous fatigue-prediction and exertion-monitoring studies using biomechanical and physiological sensor data. RF has consistently demonstrated strong performance in fatigue estimation tasks involving nonlinear, high-variance sensor inputs. For instance, Jiang et al. [29] employed RF to predict exercise-induced fatigue from IMU signals and reported robust accuracy even under limited sample conditions.

HistGBR was included to evaluate the effectiveness of boosting-based regression in capturing nonlinear feature interactions and minimizing residual errors. Although the exact histogram-based variant has not been widely used in fatigue modeling, gradient-boosting frameworks have shown strong potential for representing nonlinear dynamics in physiological signals. In particular, Cos et al. [39] compared several machine-learning algorithms and demonstrated that gradient-boosting models can effectively learn complex signal relationships in multi-sensor physiological data, while Random Forest achieved the highest overall classification accuracy for mental fatigue detection.

These findings indicate that boosting-based approaches are capable of modeling nonlinear physiological processes with high efficiency, thereby supporting the methodological rationale for adopting HistGBR in the present study.

RR, in contrast, was employed as a linear baseline model to provide an interpretable reference for comparison. By applying L2 regularization, RR effectively reduces coefficient variance and mitigates multicollinearity—common issues in physiological datasets containing correlated features such as force, EMG amplitude, and velocity.

Collectively, RF, HistGBR, and RR represent a diverse set of regression paradigms that enable a balanced comparison across model complexity, interpretability, and computational efficiency, making them suitable candidates for evaluating fatigue-related exertion.

Modeling Strategy. RF was implemented with 400 estimators and bootstrap aggregation to achieve robust performance under noisy conditions while reducing model variance. HistGBR was configured with moderate complexity (maximum depth = 6, maximum iterations = 800, learning rate = 0.04), enabling it to capture nonlinear interactions among biomechanical and engineered features. RR (α = 1.0) was included as a linear baseline model to capture global linear trends after feature standardization. This model comparison approach allowed us to investigate the relative strengths of different learning mechanisms in the context of fatigue estimation: RF provided high predictive power with low variance, HistGBR captured more subtle patterns in the data, and RR offered a computationally efficient yet less expressive baseline.

Validation and Overfitting Control. To ensure robust and unbiased model evaluation, two complementary cross-validation schemes were employed. First, a standard 5-fold K-Fold was applied to estimate baseline performance. Second, a GroupKFold strategy was implemented using participant identity as the grouping factor, thereby preventing information leakage between contralateral limbs and repeated sets from the same subject. All performance metrics were computed from out-of-fold predictions to ensure that the reported results reflected true generalization rather than overfitting.

Cut-Point Optimization. Since RPE values are discrete integers (1–10), continuous model outputs were discretized using median-based cut-point optimization. Thresholds were defined as midpoints between adjacent class medians and further constrained to maintain monotonic ordering. This approach minimized class collapse and improved the alignment of predicted outputs with the ordinal distribution of subjective exertion ratings.

Evaluation Metrics. Model performance was comprehensively assessed across multiple dimensions. Continuous prediction fidelity was evaluated using the coefficient of determination (R^2^), mean absolute error (MAE), and root mean square error (RMSE). Correlation between predicted and actual RPE values was quantified using Pearson’s correlation coefficient (r). Discrete-level performance was evaluated based on exact accuracy, tolerance-based accuracy (|error| ≤ 0.5 and ≤1.0), and confusion matrix analyses. These metrics collectively provided a detailed characterization of model behavior, error structure, and practical applicability for real-world training and rehabilitation contexts.

## 4. Results

### 4.1. Model Performance

The three models demonstrated distinct performance characteristics when applied to the fatigue estimation task. RF achieved the highest predictive performance, with an average R^2^ of 0.84, MAE of 0.62, RMSE of 0.77, and Pearson’s r of 0.91 as shown in Table 2. Furthermore, more than 93% of predictions were within ±1 RPE unit, indicating strong practical reliability.

HistGBR achieved moderate results, capturing complex feature interactions but failing to surpass RF in overall predictive fidelity. RR, while computationally efficient, underperformed relative to the nonlinear models, highlighting the importance of capturing nonlinear dynamics in fatigue-related force–time data.

### 4.2. Error Analysis

**Figure 5:**
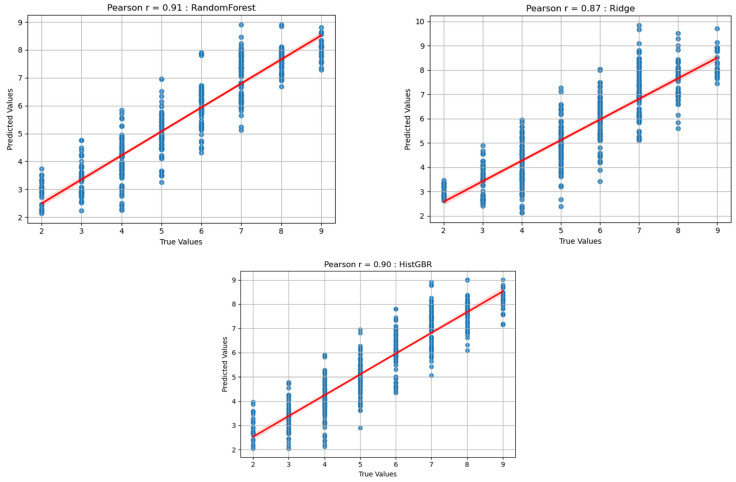
Predicted result (RF, HistGBR, RR).

Figure 5 shows the scatter plots depicting the relationships between the predicted and actual RPE values for the three regression models: Random Forest (RF), Histogram-based Gradient Boosting Regression (HistGBR), and Ridge Regression (RR). Each plot includes a fitted regression line along with the Pearson correlation coefficient (r), which quantifies the agreement between predicted and true values.

The RF model achieved the highest correlation (r = 0.91), demonstrating strong predictive accuracy and consistent performance across various fatigue levels. The HistGBR model also achieved a high correlation (r = 0.90), showing comparable performance, although with slightly greater dispersion around the regression line. In contrast, the RR model yielded a lower correlation (r = 0.87 *), indicating a limited capacity to capture the nonlinear patterns underlying fatigue progression.

These findings emphasize that nonlinear regression models (RF and HistGBR) can more effectively represent the complex force–fatigue relationships compared with the linear RR model. Such comparative results suggest that nonlinear tree-based algorithms are more suitable for modeling fatigue-related exertion under controlled isokinetic conditions.

**Figure 6:**
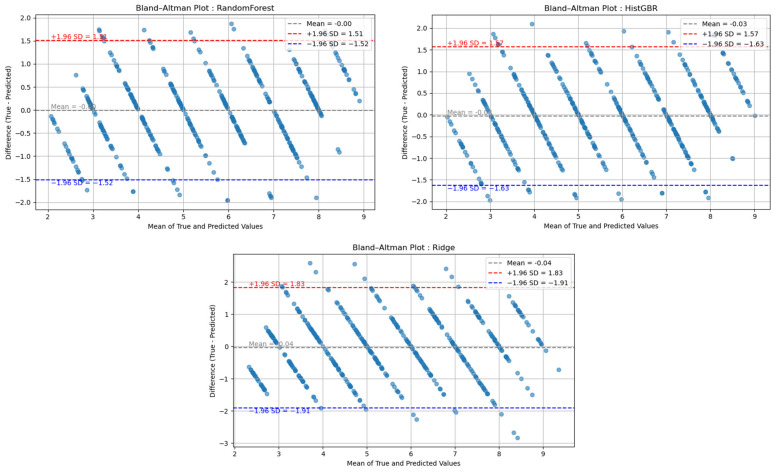
Bland–Altman (B&A) Plot (Blue = −1.96 SD, Red = +1.96 SD, Gray = Mean).

Furthermore, the error-bucket analysis confirmed that the vast majority of deviations fell within the ±1 range, with only a few cases exceeding ±2, thereby underscoring the model’s practical utility. These findings are consistent with previous fatigue prediction studies employing Bland–Altman (B&A) analysis [27,39,40], as shown in Figure 6. The results indicate that the mean difference between predicted and actual fatigue values was close to zero, and that most deviations lay within the 1.96 standard deviation (SD) limits of agreement. Specifically, participants exhibiting strong Pearson correlations showed narrow error bounds (approximately −0.5 to 0.5 units), whereas those with moderate or weak correlations demonstrated wider limits, typically ranging from ±1 to ±2 units.

Taken together, these findings reinforce that the proposed model captures subjective exertion with high fidelity, and that deviations within ±1 represent an error margin that is both statistically robust and physiologically acceptable in the context of exercise-induced fatigue monitoring.

### 4.3. Comparative Evaluation

The proposed machine learning framework was comprehensively evaluated through cross-validation to verify its predictive capability and practical applicability for fatigue estimation. Compared to baseline approaches that relied solely on raw biomechanical features or conventional regression algorithms, the developed system demonstrated significant improvements across all key metrics, including explained variance (R^2^), error measures (MAE, RMSE), and prediction accuracy within the ±1 RPE range. This performance gain was largely attributed to three main design elements: (1) the inclusion of engineered features that capture relative fatigue progression rather than static muscle output, (2) a modeling strategy capable of learning nonlinear relationships and complex interactions between biomechanical variables, and (3) a validation scheme based on group-wise cross-validation to ensure generalization across participants. As a result, the proposed approach achieved over 93% accuracy within ±1 RPE, with strong linear correlation to self-reported exertion scores, confirming its robustness and suitability for deployment in intelligent resistance training systems.

## 5. Discussion

This study demonstrates that ratings of perceived exertion (RPE) can be accurately predicted from objective force–time data when both fundamental biomechanical descriptors and carefully engineered secondary features are systematically incorporated into the modeling process. Unlike many previous approaches that treated fatigue estimation as a binary classification task (e.g., fatigued vs. non-fatigued), our method explicitly models the continuous and ordinal nature of exertion levels, allowing more nuanced and physiologically meaningful predictions.

A key finding is that RPE is more strongly associated with relative changes in performance than with absolute biomechanical values. Features representing deviations from baseline or previous sets significantly enhanced predictive performance, supporting the psychophysiological principle that perceived effort arises from changes relative to an individual’s prior capacity rather than from isolated force measurements. This aligns with established models of fatigue perception, which posit that central and peripheral signals are dynamically integrated to generate subjective exertion feedback over time.

The comprehensive feature engineering and modeling strategy employed here played a critical role in achieving high predictive accuracy. Incorporating non-linear relationships, cumulative load indicators, and temporal dynamics enabled the model to capture complex patterns of fatigue progression that conventional approaches often overlook.

Work, which represents the area under the moment-angular position curve during isokinetic testing, accounts for the overall adaptation of the curve, not solely its highest value, such as peak force [41]. Pincivero et al. proposed the suitability of a method that utilizes the total work to estimate muscle fatigue [42]. Bosquet et al. reported results suggesting that total work during isokinetic testing can be indicative of fatigue resistance and anaerobic capacity [43]. Several studies have indicated that total work measurement should be considered an important discriminator for improving the reliability of isokinetic strength tests [42,44]. In our study, the work during the bench press differed significantly between sets 1 and 7 of the exercise sets. This result indicates a significant increase in fatigue in the later stages compared to the early stages of the sets.

Features such as rate of force development (RFD) and time to peak force (TPF) pertain to the capacity for rapid force production, and these are considered to be more significant descriptors of muscle function than maximal muscle strength [45,46,47,48]. These features may be sensitively influenced by fatigue level. For instance, Grazioli et al. suggested that rapid force production capacity was significantly reduced after fatigue [49]. Zhang et al. [50] observed a notable decrease in explosive force production capacity during the initial phase of maximal contraction. However, in our study, neither RFD nor TPF exhibited significant changes across repeated exercise sets, which may be attributed to the fact that the peak force in the bench press occurs at the very beginning of the RoM (approximately the first 1–2% of RoM), rendering it seemingly unaffected by the fatigue level [51,52]. Therefore, when utilizing features such as RFD or TPF as indicators of fatigue level, it is essential to consider that each muscle’s architecture and the specific point at which peak force occurs vary [53,54].

Additionally, the use of group-wise cross-validation prevented data leakage at the subject level and ensured that performance metrics reflected true generalizability across participants.

Recent work by Molle et al. [55] established that physiotherapists heavily rely on RPE when adjusting robotic assistance during rehabilitation, yet this required repeatedly interrupting their intervention to collect subjective ratings. Our study addresses this limitation by demonstrating that RPE can be accurately predicted from force-time data alone. This automated approach enables continuous fatigue monitoring without disrupting the therapeutic flow, supporting the development of adaptive robotic systems for both clinical and home-based settings.

From an application standpoint, error distribution analyses revealed that nearly all prediction deviations fell within ±1 RPE unit, a range generally considered non-significant in practical training or rehabilitation contexts. This suggests that the model’s predictive performance is well within the thresholds of real-world usability, where small perceptual differences are unlikely to impact decision-making or load adjustments.

Nonetheless, several limitations should be noted. The current dataset was limited to healthy young male participants, potentially restricting generalizability to broader populations, including women, older adults, or clinical groups. Furthermore, only force–time signals were utilized. Future research should incorporate multimodal physiological data such as electromyography (EMG), heart rate variability (HRV), or motion kinematics to improve predictive robustness. Expanding the dataset and validating the model under free-weight or real-world training conditions will further strengthen its practical applicability and ecological validity.

## 6. Conclusions

This study demonstrated that ratings of perceived exertion (RPE) can be reliably predicted from force–time data collected during resistance training when biomechanical features are systematically combined with engineered variables that capture relative fatigue progression. By modeling exertion as a continuous and dynamic process rather than a static output, the proposed approach effectively bridges subjective perception with objective mechanical data. A key outcome of this work is the identification of relative performance decline as a more accurate and physiologically meaningful predictor of exertion compared to absolute force values.

The findings offer both theoretical and practical contributions. Theoretically, the results support psychophysiological models of effort perception, which emphasize the brain’s integration of changes in physical state over time rather than the magnitude of a single load. Practically, the proposed model provides a data-driven and interpretable framework for automated fatigue assessment, enabling intelligent resistance machines to autonomously adjust load, enhance training safety, and support personalized exercise prescription without user intervention.

Future research should aim to apply and validate the proposed model in real-world exercise environments involving diverse populations, including older adults, women, adolescents, and elite athletes. The simultaneous collection of predicted and self-reported RPE under varying conditions will help refine the model’s accuracy and improve its generalizability. Such efforts will further enhance the practical applicability of machine learning-based fatigue monitoring, ultimately contributing to the development of next-generation smart training systems that continuously adapt to individual physiological states.

Furthermore, to strengthen the model’s external validity, statistical power, and robustness, future work will involve collecting larger-scale and multi-center datasets. It is important to note that the present study was conceived as an initial, practice-oriented step toward developing real-world data acquisition strategies for large-scale applications. The finding that fatigue estimation can be achieved using force–time data alone provides a solid foundation for future integration into robotic resistance training systems, through which extensive datasets can be accumulated to enable the development of more advanced deep learning and high-dimensional modeling approaches.

There is a methodological limitation in the temporal alignment between force data and RPE labels. RPE values were collected only at the end of each set, which is standard clinical practice. This assumes that end-of-set ratings reflect the average exertion across all repetitions. Future research should explore collecting RPE values repetition-by-repetition using voice recognition or wearable interfaces to capture intra-set fatigue dynamics more accurately.

In addition, future extensions of this work will consider multimodal biosignal integration—combining physiological indicators such as EMG, heart rate, and skin conductance with force-based features—to enhance model interpretability and physiological validity. Such integration may provide a more comprehensive representation of neuromuscular fatigue processes and further improve the robustness of fatigue estimation across diverse training conditions.

Moreover, the long-term objective of this research is to integrate the proposed fatigue estimation algorithm into intelligent resistance-training systems, such as XIM, for real-time feedback and adaptive load control. The current inference latency of the model remains at approximately 20~30 ms, which is substantially shorter than the duration of a single bench-press movement—typically 1~4 s per repetition within a 30~50 cm range of motion. This ensures sufficient computational margin to complete processing between repetitions, allowing feedback to be provided immediately after each movement without perceptible delay. The algorithm’s modular, microservice-based architecture also facilitates seamless deployment into embedded hardware or robotic rehabilitation platforms. Future system-level validation will focus on confirming the stability, responsiveness, and user experience of the proposed fatigue monitoring method.

## Figures and Tables

**Table 1:** Explanation of input features.

Number	Name	Explanation
1	Peak Force	Maximum instantaneous force recorded during muscle contraction.
2	Mean Force	Average sustained force over the contraction period
3	Root Mean Square	Square root of the mean squared signal, reflecting the effective signal energy
4	Variance and Standard Deviation	Statistical measures of variability, indicating the stability of force output
5	Skewness and Kurtosis	Distributional characteristics of the force curve, describing asymmetry and peakedness of the contraction profile
6	Work	Integral of force over displacement, representing the cumulative mechanical energy produced
7	Time to Peak Force	Latency from contraction onset to maximum force generation
8	Rate of Force Development	Slope of force–time curve (ΔForce/ΔTime), representing explosive neuromuscular drive
9	Work/SD ratio	workload adjusted for stability
10	Peak × RMS	compound indicator of intensity and sustained energy
11	Δ × ratio	integrates both absolute deviation and proportional change

**Table 2:** Predictive performance comparison of machine learning models for RPE estimation.

Model	R^2^	MAE	RMSE	Pearson’s r	Within ±0.5	Within ±1
RF	0.84	0.62	0.77	0.91	0.502	0.931
HistGBR	0.82	0.64	0.82	0.90	0.487	0.929
RR	0.75	0.78	0.96	0.87	0.380	0.895

## Data Availability

Due to the proprietary nature of the data provided by the company, the dataset cannot be shared publicly. Further information may be available upon request and subject to company approval.
